# Improving Hospital Length of Stay: Results of a Retrospective Cohort Study

**DOI:** 10.3390/healthcare9060762

**Published:** 2021-06-19

**Authors:** Swapnil Patel, Abbas Alshami, Steven Douedi, Natasha Campbell, Mohammad Hossain, Arman Mushtaq, Dana Tarina, Brett Sealove, David Kountz, Kim Carpenter, Ellen Angelo, Vito Buccellato, Kenneth Sable, Elliot Frank, Arif Asif

**Affiliations:** 1 Department of Medicine, Jersey Shore University Medical Center, Hackensack Meridian Health, Neptune, NJ 07753, USA; Abbas.Alshami@hmhn.org (A.A.); Steven.Douedi@hmhn.org (S.D.); Natasha.Campbell@hmhn.org (N.C.); Mohammad.Hossain@hmhn.org (M.H.); Arman.Mushtaq@hmhn.org (A.M.); Dana.Tarina@hmhn.org (D.T.); David.Kountz@hmhn.org (D.K.); Arif.Asif@hmhn.org (A.A.); 2 Department of Cardiology, Jersey Shore University Medical Center Hackensack Meridian Health, Neptune, NJ 07753, USA; Brett.Sealove@hmhn.org; 3 Hospital Administration, Jersey Shore University Medical Center Hackensack Meridian Health, Neptune, NJ 07753, USA; Kim.Carpenter@hmhn.org (K.C.); Ellen.Angelo@hmhn.org (E.A.); Vito.Buccellato@hmhn.org (V.B.); Kenneth.Sable@hmhn.org (K.S.); 4 Department of Quality Improvement, Jersey Shore University Medical Center Hackensack Meridian Health, Neptune, NJ 07753, USA; Elliot.Frank@hmhn.org

**Keywords:** length of stay, cost savings, discharge, quality improvement

## Abstract

(1) Background: Jersey Shore University Medical Center (JSUMC) is a 646-bed tertiary medical center located in central New Jersey. Over the past several years, development and maturation of tertiary services at JSUMC has resulted in tremendous growth, with the inpatient volume increasing by 17% between 2016 and 2018. As hospital floors functioned at maximum capacity, the medical center was frequently forced into crisis mode with substantial increases in emergency department (ED) waiting times and a paradoxical increase in-hospital length of stay (hLOS). Prolonged hLOS can contribute to worse patient outcomes and satisfaction, as well as increased medical costs. (2) Methods: A root cause analysis was conducted to identify the factors leading to delays in providing in-hospital services. Four main bottlenecks were identified by the in-hospital phase sub-committee: incomplete orders, delays in placement to rehabilitation facilities, delays due to testing (mainly imaging), and delays in entering the discharge order. Similarly, the discharge process itself was analyzed, and obstacles were identified. Specific interventions to address each obstacle were implemented. Mean CMI-adjusted hospital LOS (CMI-hLOS) was the primary outcome measure. (3) Results: After interventions, CMI-hLOS decreased from 2.99 in 2017 to 2.84 and 2.76 days in 2018 and 2019, respectively. To correct for aberrations due to the COVID pandemic, we compared June–August 2019 to June–August 2020 and found a further decrease to 2.42 days after full implementation of all interventions. We estimate that the intervention led to an absolute reduction in costs of USD 3 million in the second half of 2019 and more than USD 7 million in 2020. On the other hand, the total expenses, represented by salaries for additional staffing, were USD 2,103,274, resulting in an estimated net saving for 2020 of USD 5,400,000. (4) Conclusions: At JSUMC, hLOS was found to be a complex and costly issue. A comprehensive approach, starting with the identification of all correctable delays followed by interventions to mitigate delays, led to a significant reduction in hLOS along with significant cost savings.

## 1. Introduction

In 2015, 17.8% of the United States gross domestic product (GDP) was spent on healthcare (USD 3.2 trillion), and up to 20.1% of GDP is expected to be spent by 2025 [1]. In order to combat health-related costs, momentum is building for the transformation from fee-for-service to value-based care [2]. Value-based care is a health care model that focuses on providing high-quality care with lower costs and better outcomes [3,4]. Measures to improve outcomes focus on reducing hospital readmissions, reducing adverse events, and improving patient and community engagement [3,4]. Length of stay (LOS) is a key driver of hospital costs and an indirect factor in patient safety and satisfaction; therefore, it is a key target for improvement efforts.

Jersey Shore University Medical Center (JSUMC) is a 646-bed tertiary medical center located in central New Jersey. Over the past several years, development and maturation of tertiary services at JSUMC has resulted in tremendous growth, with the inpatient volume increasing by 17% between 2016 and 2018. As hospital floors functioned at maximum capacity, the medical center was frequently forced into crisis mode with substantial increases in emergency department (ED) waiting times, ED left without treatment (LWOT), ED divert, post-anesthesia care unit (PACU) holds/delays, and a paradoxical increase in-hospital length of stay (hLOS) from 5.2 days in 2017 to 5.33 days in the first months of 2018. This increase in hLOS was persistent even after adjusting for case mix index (CMI). Prolonged hLOS can contribute to worse patient outcomes and satisfaction, as well as increased medical costs [5].

This paper reports on a quality improvement project that was designed to identify factors contributing to prolonged hLOS in our institution and design interventions to overcome these factors, assess the impact of these interventions on hLOS, and evaluate the financial impact of these interventions. To address these issues, a Capacity Management Quality Improvement Team was chartered.

## 2. Materials and Methods

### 2.1. Overview of the Project

This project specifically aimed to decrease the CMI-adjusted hLOS by 0.5 days while improving outcomes and patient satisfaction. The project started in 2018, yet full deployment of included interventions was not complete until mid-2019. We utilized the Standards for Quality Improvement Reporting Excellence (SQUIRE) 2.0 guidelines to report this project [6].

### 2.2. Context

Jersey Shore University Medical Center (JSUMC) is a 646-bed tertiary teaching medical center in central New Jersey with seven residency and six fellowship programs. Excluding psychiatry and women’s and children’s services there are 413 beds, including 62 critical care beds, 7 medicine/surgery units containing 81 beds, and 7 telemetry units containing 270 beds.

### 2.3. QI Process

**Figure 1:**
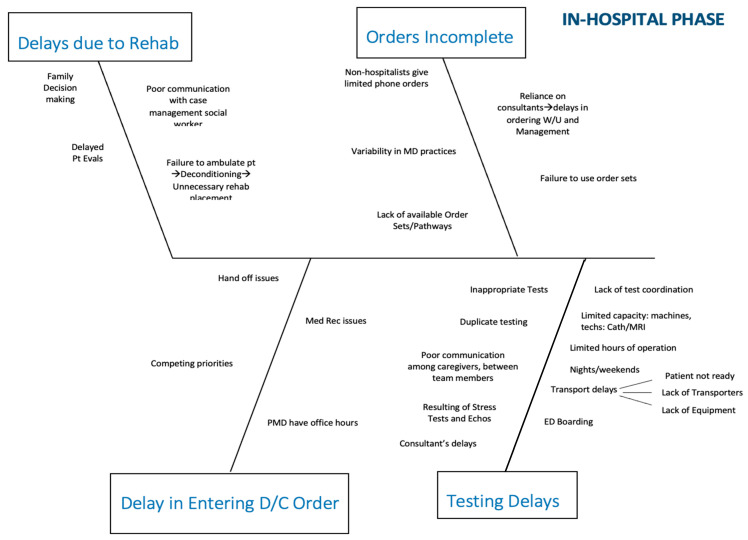
Fishbone diagram representing in-hospital phase delays, including orders and testing.

**Figure 2:**
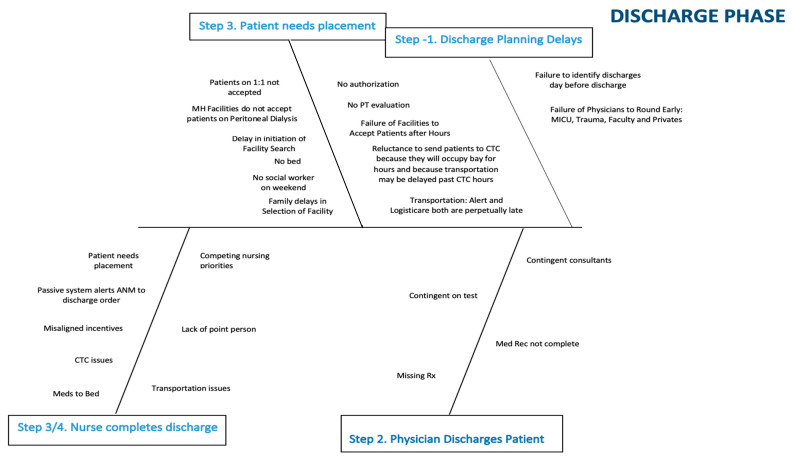
Fishbone diagram representing discharge planning and placement delays.

In April 2018, we established a multi-disciplinary team to address capacity management. The team, sponsored by senior leadership, included representatives from the medical staff, the medical residency program, front-line nursing, nursing leadership, process improvement/quality, environmental services, imaging, cardiac services, and operations. The team formed sub-committees to address issues related to the emergency department (ED)/admission, in-hospital, and discharge phases of the process. It became quickly apparent that most of the ED issues were related to bottlenecks further downstream and that addressing in-hospital and discharge issues would be most productive. These two sub-committees used root cause analysis to identify the factors leading to delays in throughput. The sub-committees were encouraged to continually ask “why?” as they drilled down on obstacles to patient flow. Every idea put forth was cataloged and then prioritized by the sub-committees. Four main bottlenecks were identified by the in-hospital phase sub-committee: incomplete orders, delays in placement to rehabilitation facilities, delays due to testing (mainly imaging), and delays in entering the discharge order. Similarly, the discharge process itself was analyzed, and obstacles were identified. For each major issue, potential contributing factors were isolated and represented in fishbone diagrams (Figure 1 and Figure 2).

### 2.4. Interventions

Once consensus was reached on the major obstacles to throughput, the committees brainstormed potential solutions to the identified obstacles. These potential solutions were tabulated as shown in Table 1 and Table 2. Interventions were then prioritized to develop a “LOS bundle”. Key elements of the bundle included encouraging use of the hospitalist service model, improved staffing in select areas, deploying mid-level providers for non-teaching patients, creating standardized multidisciplinary rounds, adopting an early ambulation program, and improving ancillary services turnaround times (TATs) for echocardiography, stress testing, ultrasonography, and MRI.

### 2.5. Increase Use of Hospitalist Model of Care

The concept of a dedicated physician that assumes responsibility for managing the patients during their hospital stay and hands off their care to primary care physicians upon discharge, or the hospitalist, was introduced in the United States a few decades ago. Since then, the hospitalist model has been associated with high-quality and efficient care and, thus, is being increasingly utilized by healthcare systems [7]. At JSUMC, at the start of this project, approximately 30% of med/surg patients outside the critical care units were managed by the hospitalists team. This team had demonstrated stellar outcomes and had begun to show improvement in efficiency with an actual/expected mortality ratio of 0.28 and hLOS significantly better than their peers (5.67 days vs. 6.05). Therefore, most patients who were admitted to the hospital through the ED and did not have a primary care physician (PCP) who would attend them in the hospital were assigned to the hospitalist service. In addition, outreach efforts to encourage PCPs to admit to the hospitalists and for surgeons to adopt co-management arrangements were undertaken. Hospitalist staffing was gradually increased to preserve an optimal physician/patient ratio.

### 2.6. Deployment of Mid-Level Providers

While 30% of adult medical/surgical patients were managed by hospitalists and an additional 30% were managed by residents in conjunction with faculty, approximately 40% of non-ICU beds were managed by private medical providers (PMDs). These patients were frequently reported to have incomplete admission orders, delayed discharges to conform to office hours, and significant variability in practice. Therefore, a mid-level provider (Advanced Practice Nurse or Physician Assistant) was hired to augment care on each med/surg/telemetry unit. Mid-levels were asked to prioritize patients who were over 65 or had complex social or medical conditions and to focus particularly on those not managed by hospitalists or residents. While still evolving, their mission was to facilitate coordination of care by ensuring complete admission orders, communication with consultants, and interfacing with social workers to prevent delays in discharges. In addition, they were tasked with quality initiatives such as ensuring the necessity and documentation for patient restraints, intravenous lines, and bladder catheters.

### 2.7. Early Ambulation Program

Lack of ambulation, especially in elderly patients, during hospitalization can result in functional decline and deconditioning [8]. Muscle deconditioning can be noticed as early as 72 h with lack of activity [9]. In addition to being an unnecessary negative patient outcome in and of itself, deconditioning will frequently necessitate placement in a rehabilitation facility after discharge. We observed that patients transferred to rehabilitation facilities have an hLOS of 2–3 days longer than those discharged home due to the transfer, placement, and authorization processes. Therefore, as part of this initiative, a nurse-driven early ambulation program was approved by the Medical Executive Committee of the hospital and implemented, with nurse managers accepting ownership of the process. Using a previously validated protocol (need reference), nurses were empowered to get patients out of bed and walking regardless of the initial activity order. We identified two obstacles to early mobilization: a nursing culture very focused on fall prevention that relied heavily on physical therapy evaluation prior to mobilizing older patients, and a physician culture of defaulting to a bed rest order on admission. To help facilitate a culture change, we performed organization-wide education on the importance of early mobilization and risks of unnecessary immobilization, achieving buy-in from the medical staff to allow for nurse discretion.

### 2.8. Ancillary Services

The increased utilization of imaging and other ancillary services in the management of patients can lead to overcrowding and significant delays in these services, which ultimately can lead to a prolonged hospital stay. At JSUMC, we found that echocardiography, stress testing, ultrasonography (US), magnetic resonance imaging (MRI), and physical therapy services had significant room for improvement to aid in our goal to decrease LOS.

#### 2.8.1. US

Prior to intervention, TAT for US was 14–16 h, with a significant number of studies that were left for the next day. An additional US technician was hired for the evening shift to meet the daily demand.

#### 2.8.2. MRI

Prior to intervention, MRI services were provided from 7 a.m. to 11 p.m. with two scanners operational 6 days a week and one scanner on Sundays, a TAT of 19 h. A steady growth in MRI utilization suggested that TAT would continue to increase. In addition, as a Comprehensive Stroke Center and Trauma Center, there was a quality imperative to improve MRI availability. Therefore, staffing was secured to allow expansion of inpatient MRI operation hours to 24/7. In addition, a second scanner was utilized on Sundays from 11:00 to 19:30 to prevent delays during weekends.

#### 2.8.3. Stress Testing

Prior to intervention, all stress tests were directly supervised by a cardiologist attending. TAT for stress testing was excellent during the weekdays. However, there was limited testing on Saturdays and no testing on Sundays. Furthermore, dependence on in-room supervision by cardiologists required staffing and operation of two stress labs despite low volume to accommodate cardiologists’ schedules. Based on previous research, the utilization of advanced nurse practitioners (APNs) in stress testing is a safe and reliable practice [10,11,12]. Therefore, 1.5 full-time APNs were employed to perform stress tests 7 days/week. A cardiologist or a cardiology fellow is immediately available to support the APN.

#### 2.8.4. Physical Therapy

Prior to intervention, physical therapy was available only on weekdays leading to delayed discharges, especially to skilled nursing or rehabilitation facilities. As a result, there was a TAT of 1.1 days (aggregate) and 1.34 days on Mondays. Therefore, 1.0 and 0.4 full-time physical therapists were employed to cover weekdays and weekends, respectively.

### 2.9. Outcomes Measures

Due to seasonal variations in the number and severity of hospitalizations of several conditions [13,14,15,16], an annual average case mix index adjusted length of stay (CMI-LOS) was used as a primary outcome measure to determine the efficacy of all interventions. CMI is defined as the average relative weight of diagnosis-related groups (DRGs) [17]. CMI-LOS was calculated by dividing the average LOS by the CMI. In 2020, especially from February to May, the geographical areas served by JSUMC were devastated by the pandemic of coronavirus 2019 (COVID-19). Patients with COVID-19 tend to stay longer in the hospital, thus potentially impacting LOS and underestimate the effects of the interventions [18]. Therefore, for this analysis, we compared CMI-LOS during June–August of 2020, when the number of patients with COVID-19 was relatively low, to the same months from 2019. In addition, we used CMI-LOS for June–August 2020 to project CMI-LOS for the full year, as if the COVID-19 pandemic did not occur. For ancillary services, we also measured turnaround time (TAT), which is the time from request until completion, during the relevant time periods.

## 3. Results

### 3.1. Length of Stay

**Figure 3:**
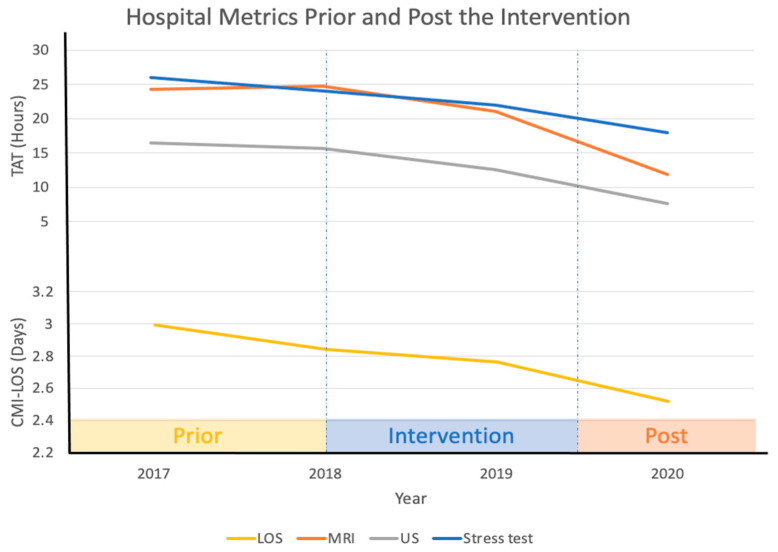
Pre- and post-intervention hospital metrics. Abbreviations: turnaround time (TAT); case mix index (CMI); length of stay (LOS); magnetic resonance imaging (MRI); ultrasound (US).

Prior to the interventions, CMI-LOS was 2.99 (1.93–4.05) days in 2017. Post-intervention median CMI-LOS decreased to 2.84 (1.81–3.86) and 2.76 (1.71–3.81) days in 2018 and 2019, respectively. The overall CMI-LOS during the first nine months of 2020 was 2.76 (1.71–3.82) days. Using the Mann–Whitney U test, the reduction from 2017 to 2018 was not significant (*p* = 0.19); however, the reduction from 2017 to 2019 and to 2020 was significant (*p* < 0.01 for both). CMI-LOS was 2.67 days during Jun–Aug of 2019 (which represents 96% of average CMI-LOS of 2019) and decreased to 2.42 days during the corresponding months of 2020. If the COVID-19 pandemic had not happened in 2020, we estimate that the CMI-LOS would have been 2.52 days (Figure 3).

### 3.2. Financial Impact

With a projected reduction of 0.5 days of CMI-hLOS had the COVID-19 pandemic not happened and an estimated CMI of 1.8 in 2019, we estimate the reduction in overall hLOS to be 0.9 days. A conservative estimate is that the hospital would save USD 500 for each full day reduction in hLOS. This translates to monthly savings of USD 625,000. The projected savings in 2020, absent of COVID-19, therefore, is USD 7.5 million. We estimate that the intervention led to an absolute reduction in costs of USD 3 million in the second half of 2019 and more than USD 7 million in 2020. On the other hand, the total expenses, represented by salaries for additional staffing, were USD 2,103,274, resulting in an estimated net saving for 2020 of USD 5,400,000. Given that the hospital was operating at maximum capacity, we believe these interventions also allowed us to accommodate continued growth. While we did not formally assess how much growth truly represented patients that would have been accommodated elsewhere, any program that allows for continued growth without building new beds will result in increased revenues averaging USD 15,000 per admission. If just one new patient per day is admitted this would translate into over USD 5,000,000 of annual revenue.

## 4. Discussion

Jersey Shore University Medical Center (JSUMC) has experienced tremendous growth over the past three years. Inpatient volume, which was 24,783 in 2016, exceeded 29,000 in 2018, an increase of 17%. With a similar growth in observation patients, our total “Heads-in-Beds” will approach 40,000. During this period, our institution only has added 11 beds and 10 recovery bays to accommodate our increasing med/surg volume. We also gained 12 beds in pediatrics and 4 labor and delivery rooms. However, despite the additional beds in various departments, this was a modest increase to accommodate our increased patient volume. Therefore, improving hLOS became vital to our institution.

For facilities at or near capacity, reducing hLOS also can enhance revenue and improve operational effectiveness. Improvement in operational effectiveness, by decreasing ED boarding, PACU delays, and ICU placement delays, can directly translate into improvement in patient satisfaction and clinical outcomes. In addition, the US Department of Health and Human Services enlisted value-based care as one of four priorities [19]. Hospitals in the United States are expected to receive significant economic penalties if they do not adhere to the value-based care model [20]. Solutions that improve quality of care and decrease costs simultaneously, such as reduction of hLOS, are imperative.

The present study is limited in that it is observational in nature, and other contributing factors could have contributed to the improvements observed. It is also possible that improved documentation led to an increase in CMI, thereby leading to an overestimation of the reduction in CMI-adjusted LOS. However, a robust clinical documentation program was in place prior to the start of this project, and no formal efforts to alter that program occurred during the study period.

## 5. Conclusions

It was clear to our team that hLOS is a complex issue. Therefore, single interventions or interventions focused on one aspect of care were unlikely to significantly decrease hLOS. A comprehensive approach, starting with extensive identification of all correctable delays followed by interventions to mitigate delays, was more likely to achieve an impactful change. We believe that this methodology—careful analysis of underlying causes by a team representing all relevant stakeholders, especially front-line staff—is applicable to many of the challenges faced in the complex healthcare environment. We also believe that many of the factors we identified and solutions we deployed are likely to be common to other institutions facing throughput challenges.

## Figures and Tables

**Table 1:** Fishbone for in-hospital phase crosswalk. Abbreviations: advanced practice nurse (APN); multi-disciplinary rounds (MDR); discharge (D/C); physical therapy (PT).

Obstacle	Potential Solutions
**Orders Incomplete**	
Non-hospitalists give limited phone orders	Increase use of hospitalists/unit-based APNs
Variability in physician practices	Hospitalists/APNS/MDR rounds
Failure to use order sets	APNs
Lack of available order sets/care pathways	Develop orders sets/pathways
**Testing Delays: Physician Related**	
Inappropriate tests: not indicated	APNs/MDR
Inappropriate tests: Could be done as O/P	APNs/MDR
Duplicate testing	APNs/MDR
Consultant-related order delay	APNs
Poor communication among team members	MDR/APNs
Delayed results: echo and stress	Remote reading/APN stress model
**Testing Delays: System Related**	
Wasted transport time due to poor test coordination	Command center
**Limited Capacity/Machines, Techs, Hours**	
MRI (hours)	Staff MRI 24/7
Cath lab (rooms/staff)	Improve capacity and efficiency
Ultrasound (hours)	Increase evening staffing
Stress test (weekend hours)	Institute APN stress model
**Transport Delays**	
Patient/nurse not ready	Command center
Lack of transporters	Staffing issue
Lack of equipment	Purchase wheelchairs/stretchers as needed
**Delayed Entry of D/C Orders**	
Competing priorities	MDR/APNs
PMD rounds AFTER office hours	APNs
Handoff issues (Weekends)	MDR/APNs
**Delays 2/2 Rehab Placement**	
Failure to ambulate	OOB initiative
Poor communication among team members	MDR/APNs
Delayed PT evaluation	Increase PT staffing
Family decision making	MDR/APNs

**Table 2:** Fishbone for discharge phase crosswalk. Abbreviations: advanced practice nurse (APN); multi-disciplinary rounds (MDRs); Meridian Health (MH); social worker (SW); assistant nurse manager (ANM); care transition center (CTC); prescription (Rx).

Obstacle	Potential Solutions
**Discharge Planning Delays**	
Failure to identify discharges until day of discharge	MDR
**Placement Issues**	
Patients on 1:1 observation not accepted	None
MH facilities do not accept PD patients	Work to change policy
Delay in initiation of facility search	MDR/APNs
Lack of SW support on weekends	Increase SW resources on weekends
Delays in authorization	Have facilities accept patients pending authorization
Failure of facilities to accept patients after hours	Have standard set of rules for all participating facilities
Transport Delays: Alert	Have alternative provider available
Transport Delays: LogistiCare	None
**Nursing Delays**	
Misaligned incentives	Create incentives for nurses to discharge early
Passive alert system	Discharge board
Meds to beds	Should not be an obstacle (can deliver to CTC or lounge)
Competing nursing priorities	ANM to assist
Lack of ownership of process	Nurse manager or designee to “own” process
Internal transportation issues	Rapid discharge team
**Clinical Delays**	
Delayed physician discharge order	Hold hospitalists, faculty, residents accountable
	APN deployment
Med Rec and Rx issues	Increase use of hospitalists/APNs

## Data Availability

All data used for this study are available within the manuscript.
